# Moyamoya disease in a 10-year-old male patient in the Middle East with the outcome of the surgery: A case report and literature review

**DOI:** 10.1016/j.ijscr.2025.111557

**Published:** 2025-06-23

**Authors:** Imad Talahma, Saja E. Abusabha, Razan M. Abu Ali, Marwa J. Hrainy, Omar Kh. Sarahna, Mohammad F. Nu'man

**Affiliations:** a Neuro-spine Palestine Center, Alhilal Speciality Hospital, Hebron 90200, Palestine; b Faculty of Medicine, Palestine Polytechnic University, Hebron 90200, Palestine

**Keywords:** Moyamoya disease, Pediatrics, Indirect revascularization, Palestine

## Abstract

**Introduction:**

A chronic cerebrovascular illness called Moyamoya disease (MMD) causes the internal carotid artery and the circle of Willis to narrow. Children can benefit from indirect bypass surgery, a procedure that is widely practiced in East Asia. It's critical to address postoperative problems early.

**Presentation of case:**

A 10-year-old male presented with syncope and loss of consciousness at the age of 9 years, followed by headaches, slurred speech, and aphasia. Imaging revealed acute ischemia in the left MCA territory. Lab tests showed coagulation abnormalities, and he was referred for stroke treatment. Eleven months later, he had another episode with headaches and vomiting; imaging showed chronic ischemic damage and a new infarction in the right temporal lobe. He underwent indirect revascularization but deteriorated postoperatively, developing hypoactivity, aphasia, and limb weakness. A CT scan indicated new ischemic changes. Six months before the last visit, he presented with tetraparesis, dysarthria, and blindness and was referred for rehabilitation.

**Discussion:**

Moyamoya disease is a rare cerebrovascular condition characterized by cerebral ischemia or hemorrhage. The condition is linked to thinning of blood vessel walls, decreased blood flow, and neurological symptoms like speech loss or weakness. It is characterized by intracerebral internal carotid artery stenosis and abnormally produced collateral arteries. Cerebral cortical artery revascularization is the primary surgical treatment, but it can also cause other unusual symptoms.

**Conclusion:**

In Palestine, Moyamoya disease is uncommon and needs to be treated right away. It's critical to monitor stroke following surgery and take MMD into account when making a differential diagnosis for neurological symptoms in children.

## Highlights

• Rare case in Palestine: A toddler from Palestine developed Moyamoya disease (MMD) in a rare case that was made worse by a stroke following surgery.
• After indirect revascularization surgery, the patient experienced a stroke three days later.
• Pediatric MMD: This article draws attention to the necessity of considering MMD when a child exhibits neurological symptoms that cannot be explained.
• Operative strategy: Indirect revascularization was the first therapy strategy, and it usually works well for children.

## 1 Introduction

Moyamoya disease (MMD) is a chronic cerebrovascular condition that causes the progressive narrowing of the circle of Willis and the terminal portion of the internal carotid artery. This results in the development of a fragile collateral vascular network at the base of the brain [1]. MMD is most prevalent in East Asia, with a slight predominance of occurrence in females [2]. According to epidemiological studies carried out in Japan, there are between 3.2 and 10.5 instances per 100,000 people [3]. Surgical interventions such as indirect bypass are effective treatments for MMD. In most cases, children respond well to indirect bypass, and it is uncommon for children over three years of age to experience new cerebral infarction at the surgical site following the indirect method of surgery [4]. Our study focuses on a 10-year-old male who presented with Moyamoya disease and was complicated by a stroke three days after the indirect revascularization surgery. This report aims to shed light on this rare entity. It emphasizes the importance of not underestimating Moyamoya disease and the risk of developing early complications after surgery in pediatrics with this disease. This article has been reported in line with the SCARE 2025 guideline, which updates the consensus Surgical Case Reports (SCARE) guidelines [5].

## 2 Presentation of case

**Fig. 1:**
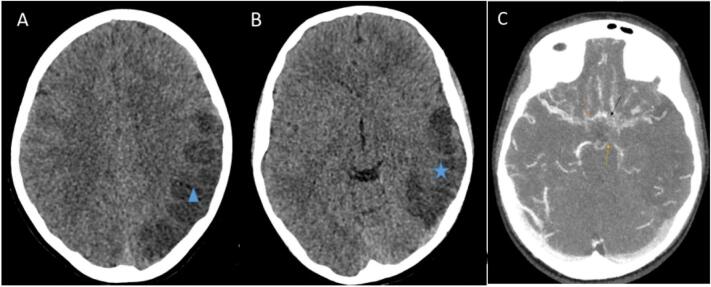
Selected axial brain CT scan images were done for the same patient, Brain CT scan without contrast (A + B) and brain CT angiography with MIP (maximum intensity projection) technique. They are showing left parieto-occipital (blue triangle in an A image) and temporal (blue star in B image) cortical and subcortical low attenuating area denoting subacute ischemic injury in the territory of left MCA (middle cerebral artery) and left PCA (posterior cerebral artery). (C) Image shows a complete cutoff at M1 segment of the left middle cerebral artery (black arrow) and narrowing at M1 segment of the right MCA (orange arrow) and left PCA (yellow arrow).

**Fig. 2:**
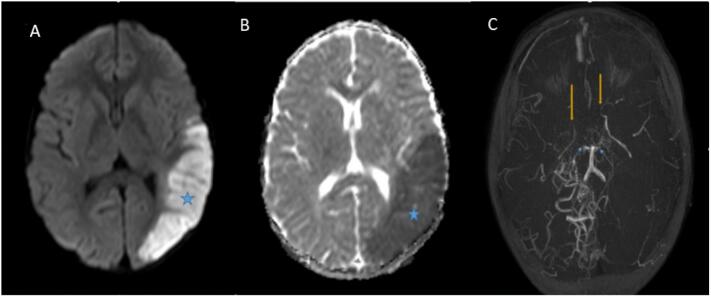
Selected axial brain images for our patient, Brain MRI and MRA (A) diffusion sequence demonstrates Left parieto-occipital cortical and subcortical area of high signal (blue star) with corresponding image low signal seen at (B) ADC sequence (Apparent diffusion coefficient) (blue star) in keeping with acute infarction. In MRA image (C) There is a significant narrowing of both middle cerebral arteries (MCA) M1 segments (yellow arrows) and a narrowing of P1 segments of both posterior cerebral arteries (PCA) (blue arrows).

**Fig. 3:**
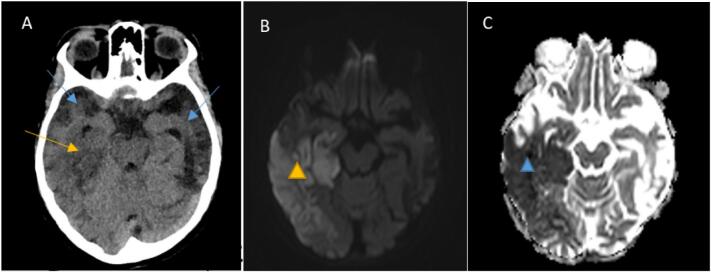
Selected axial brain images for our patient, brain CT scan Brain MRI (A) axial brain CT scan cut is showing bitemporal lobe atrophy (blue arrows) as well as right posterior temporal lobe hypodensity suggestive of early ischemic changes. Corresponding MRI images, (B) diffusion sequence demonstrates right temporoparietal cortical and subcortical area of high signal (yellow triangle) with corresponding image low signal seen at (C) ADC sequence (Apparent diffusion coefficient) (blue triangle) in keeping with acute infarction.

**Fig. 4:**
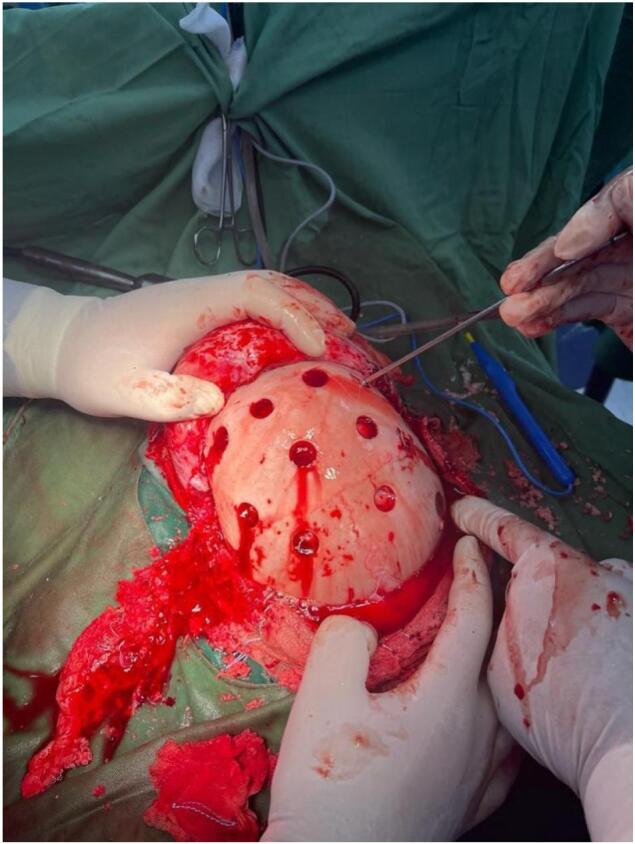
Interoperation pictures showing multiple burr holes drilled over the exposed areas of bone, through small incisions in the periosteum to place a muscle layer onto the brain's surface to encourage blood vessels to grow into the ischemic brain.

**Fig. 5:**
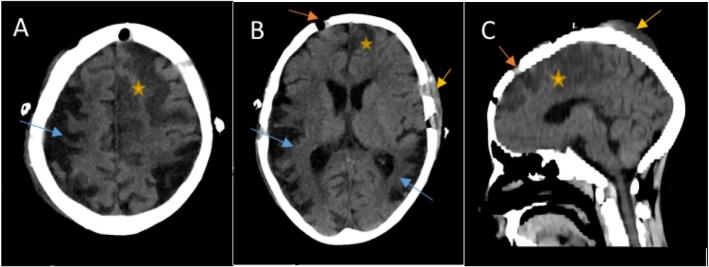
Selected axial brain CT scan images were done for our patient after surgery. They are showing new left frontoparietal (yellow stars in all images) cortical and subcortical low attenuating area denoting subacute ischemic injury in the territory of left ACA (Anterior cerebral artery). Blue arrows in A and B images refer to encephalomalacia areas secondary to old infarctions. Postoperative changes are noted as yellow arrows in B and C images are related to subgaleal hematoma and subcutaneous edema at site of surgery. While orange arrows at image same images show multiple skull bone defects.

A 10-year-old male patient otherwise healthy with no significant past medical history, initially presented to the emergency department in a private hospital two years ago at the age of 9 years with syncope and loss of consciousness for a few seconds that happened 1 h after falling. These symptoms were associated with headache, tongue deviation, slurring of speech, and difficulty hearing, suggestive of Broca's and Wernicke's aphasia. There was no history of fever, seizure, visual disturbances, or vomiting. No family history of early stroke or ischemic heart disease was reported. On examination, he was confused, but his vital signs were stable, and his muscle power was 5/5 using the Medical Research Council (MRC) muscle strength scale. He underwent a computed tomography angiogram (CTA) of the brain with contrast that showed evidence of acute early parietooccipitotemporal ischemia with a complete cut-off at the level of the left middle cerebral artery (MCA) (Fig. 1). Brain magnetic resonance imaging (MRI) and magnetic resonance angiography (MRA) showed that there was a hyperintensity on diffusion-weighted imaging with corresponding hypointensity on Apparent diffusion coefficient (ADC) at the left temporoparietal region in the territory of the left MCA, representing an acute ischemic stroke (Fig. 2). Laboratory investigations showed a mild elevation in prothrombin (factor II) and protein C concentrations and a decreased factor VIII concentration. A hematological consult was made for stroke treatment, and he was instructed to follow up with a pediatric neurologist. Even though the patient first showed signs of an acute ischemic stroke at the age of 9, the diagnosis of Moyamoya disease was not officially made at that time; it was made retrospectively following a second ischemic episode at the age of 10. Neuroimaging showed progressive bilateral stenosis of the internal carotid and middle cerebral arteries, along with the development of abnormal collateral vessels, which are the hallmark features of Moyamoya disease. 11 months later, the patient presented again to the emergency department, complaining of a new-onset headache and vomiting. On examination, he was afebrile, well, conscious, and oriented. A computed tomography (CT) scan without contrast showed a bilateral cortical encephalomalacia involving the cortex of both parietal lobes, representing a chronic ischemic insult, in addition to a hypodense area at the right temporal lobe, MRI was done which showed that he had an acute infarction at the right temporoparietal lobes posteriorly at the territory of the MCA inferior branch (Fig. 3). Due to institutional limitations and lack of patient consent for further imaging, the precise angiographic images of the internal carotid artery (ICA) and external carotid artery (ECA) are unavailable, which restricts the completeness of radiological data in this report. Indirect revascularization was selected due to the patient's youth and the surgical team's familiarity with indirect methods. Angiogenesis is more active in children, and indirect techniques often yield favorable results. Additionally, direct bypass was not feasible due to institutional limitations and technical constraints. The patient was then informed to undergo an indirect revascularization procedure (frontotemporal per hole with temporal craniotomy and temporal muscle insertion over brain tissue) (Fig. 4). Postoperatively, he was discharged in a good status; active, alert, complaining of no bleeding, and was advised to follow up in the neurosurgery clinic after a week. On the third day post-operation, the patient suddenly became hypoactive and aphasic with decreased power and tone in all his extremities, for which he was transferred to the Pediatric Intensive Care Unit (PICU) at the same hospital. CT scan without contrast showed subgaleal hematomas up to 1.3 cm thick and mild subgaleal inflammatory edema. Multiple foci of confluent cortical, subcortical, and deep white matter hypodensities suggesting encephalomalacia and ischemic changes were present, likely indicating a new stroke (Fig. 5). These symptoms were most likely caused by a postoperative ischemic stroke affecting the anterior cerebral circulation, particularly the left ACA region. The pathophysiology likely involved watershed infarction or hypoperfusion due to insufficient collateral formation following the indirect procedure. At the most recent visit 6 months ago the patient presented with tetraparesis, dysarthria, and blindness. He was then referred to a physiotherapist in a specialized center. Despite the need for further diagnostic evaluation, socioeconomic and logistical constraints prevented the family from pursuing additional investigations. The clinical team prioritized supportive care and rehabilitation due to the severity of neurological impairment. He was referred to physiotherapy to address his significant functional deficits in speech, mobility, and daily living. Early rehabilitation is crucial in pediatric stroke cases to prevent complications and maximize neuroplastic recovery.

## 3 Discussion

Moyamoya disease is a rare cerebrovascular illness that primarily manifests as signs and symptoms of cerebral ischemia or cerebral hemorrhage with a generally progressive course [2]. This disease is identified by intracranial internal carotid artery stenosis or occlusion and subsequent abnormally formed collateral vessels [6]. The incidence rate in the Middle East is still unknown [3]. The pathologies associated with MMD include the thinning of the middle layer of the blood vessel wall, the reduction of the outer diameter of the blood vessel, irregular growth of the inner elastic layer, and intimal fibrous hyperplasia of the cerebral artery stenosis [7]. Recently, it was found that affected siblings had specific human leukocyte antigen markers on chromosome 6 [8]. There are two main types of clinical symptoms associated with MMD: cerebral hemorrhage and cerebral ischemia. In most pediatric patients, there is an increase in cerebral ischemia, cerebral infarctions, and transient ischemic episodes upon arrival [7]. MMD typically leads to reduced blood flow in the region of the internal carotid artery, causing localized neurological symptoms like weakness on one side of the body, difficulty speaking, or loss of speech. However, it can also cause other unusual symptoms such as fainting, weakness in the lower body, eye problems, or involuntary movements, especially in children [9]. The main surgical procedure in MMD is cerebral cortical artery revascularization. This can be accomplished through direct revascularization (connecting scalp arteries directly to cerebral cortical arteries), indirect revascularization (placing a scalp or muscle layer onto the brain's surface to encourage blood vessels to grow into the ischemic brain), or a combination of both methods [4]. Indirect revascularization was chosen in this case due to the patient's age and institutional familiarity with the procedure. It is known to be more effective in children due to their angiogenic capacity, though it requires time for collaterals to form. In our literature, there is only one case discussing Moyamoya disease in children in Palestine [3]. This is the second pediatric Moyamoya case documented in Palestine and one of the few reported in the Middle East, highlighting its geographical relevance. It also offers insights into the challenges of managing such cases in resource-limited settings, including delayed diagnosis due to limited access to pediatric neurology and neurosurgical care. According to data from the cohort study, patients undergoing indirect revascularization had a significantly lower risk of stroke occurrence during follow-up, even though they presented with more severe symptoms than patients receiving conservative treatment [10]. Our case, however, resulted in a poor outcome, and several contributing factors may explain this:

• The patient had advanced bilateral disease at the time of surgery, which is associated with a poor prognosis.
• There was a significant delay (11 months) between symptom onset and surgery, allowing more ischemic damage to occur.
• A stroke occurred before new collateral vessels had the chance to form post-surgery.
• Coagulation abnormalities—including elevated prothrombin and protein C, and decreased factor VIII—may have contributed to impaired cerebral perfusion and thrombotic risk.
• The combination of extensive disease, delayed intervention, perioperative ischemic insult, and hematological risk factors likely led to the severe neurological deficits observed.
• Literature from other centers such as Korea and North America using similar techniques demonstrates generally positive outcomes with indirect revascularization, especially when performed early [4,10]. However, the success of surgery heavily depends on timing, perioperative care, and patient-specific risk factors.

During the last six months of follow-up, the patient's family faced logistical and socioeconomic challenges that prevented further diagnostic investigations. Given the patient's severe neurological impairments, rehabilitation and supportive management were prioritized.

## 4 Conclusion

Moyamoya disease is rare, especially in Palestine. MMD should be considered in the differential diagnosis of any neurological symptom in children and may indicate a rare and serious condition that requires immediate intervention. Surgical treatment reduces the incidence of stroke, but follow-up should be done to ensure that it does not occur in any age group. This case highlights the importance of early recognition, appropriate surgical planning, and close postoperative monitoring. It also underscores the multifactorial challenges faced in managing Moyamoya disease in low-resource settings, where diagnostic delays and limited access to care can adversely affect outcomes.

## CRediT authorship contribution statement

Saja E. Abusabha, and Razan M. Abu Ali: Conceptualization, case analysis, manuscript writing, and editing.

Omar Kh. Sarahna, and Marwa J. Hrainy: Data collection, literature review, and manuscript drafting.

Mohammad F. Nu'man: Supervision.

Imad Talahma: Clinical management of the patient, data interpretation, and manuscript revision.

## Patient consent

Written informed consent was obtained from the patient's mother to publish this case report.

## Ethical approval

This case report is exempt from ethical approval in our institution.

## Funding

No sources of funding for this case report.

## Declaration of competing interest

The authors have no conflict of interest to declare.

## Data Availability

The data used to support the findings of this study are included in the article.
